# Psychophysiological Responses of Exercise Distribution During High Intensity Interval Training Using Whole Body Exercise

**DOI:** 10.3389/fphys.2022.912890

**Published:** 2022-08-22

**Authors:** Alexandre F. Machado, Paulo Vinicios Camuzi Zovico, Alexandre L. Evangelista, Roberta L. Rica, João Marcelo de Q. Miranda, Cristine Lima Alberton, Valentina Bullo, Stefano Gobbo, Marco Bergamin, Julien S. Baker, Danilo S. Bocalini

**Affiliations:** ^1^ Laboratory of Experimental Physiology and Biochemistry, Federal University of Espírito Santo, Physical Education and Sport Center, Vitoria, Brazil; ^2^ Ibirapuera University, São Paulo, Brazil; ^3^ Department of Physical Education, Estacio de Sá University, Vitoria, Brazil; ^4^ Department of Physical Education, Cidade de São Paulo University, São Paulo, Brazil; ^5^ Department of Sports, Federal University of Pelotas, Pelotas, Brazil; ^6^ Department of Medicine, University of Padova, Padova, Italy; ^7^ Center for Health and Exercise Science Research, Department of Sports, Physical Education and Health, Hong Kong Baptist University, Kowloon Tong, China

**Keywords:** bodyweight, exercise, exercise order, psychophysiological, HIIT body work, training

## Abstract

The time-efficient nature of HIIT using bodyweight exercises can facilitate the application of exercise programs at home by encouraging more people to perform regular physical exercise. However, there are no studies investigating the influence of the distribution/order of exercises during HIIT training sessions using this method. The aim of the present study was to evaluate the effects of different exercise orders on training load indicators during HIIT sessions using body weight. Twenty male participants performed three 20-min sessions of HIIT using whole body exercise, consisting of 20 sets with 30 s of activity performed at maximal intensity, followed by 30 s of passive recovery. Three designs of exercise protocols were randomly performed according to the following exercise distribution: A: jumping jack, burpee, mountain climb and squat jump); B: jumping jack, mountain climb, burpee, and squat jump) and C: burpee, squat jump, jumping jack and mountain climb. No differences were found between protocols for relative heart rate, perceived exertion, and lactate concentrations. Significant differences (*p* < 0.001) were found for the number of movements (A:712 ± 59, B:524 ± 49, C:452 ± 65). No differences were observed for the area under curve when examining perceived exertion between protocols. However, the values for perceived recovery significantly differed (*p* < 0.001) between protocols (A:64 ± 19; B:52 ± 11; C:17 ± 13). Interestingly, protocol B and C induced a displeasure perception compared to protocol A. Our findings suggest that exercise distribution/order using HIIT whole body exercise promotes alterations in psychophysiological responses in HIIT using whole body exercises.

## 1 Introduction

High intensity interval (HIIT) training has been considered as an effective method to increase maximum oxygen uptake (VO_2_), time to exercise exhaustion, maximal activity of cytochrome c oxidase (COX) and total protein content of peroxisome proliferator-activated receptor coactivator one alpha (PGC-1α). These increases contribute to improvements in cardiometabolic parameters (Gibala and McGee, 2008; Nogueira et al., 2012), decrease body fat (Alves et al., 2017; Khammassi et al., 2018) and promote increases in the functional capacity of practitioners (Gibala et al., 2012).

A potential alternative complementary training regime using HIIT is bodyweight training, currently ranked in seventh position in the American College of Sports Medicine (ACSM’s) Fitness Trends (Thompson, 2019). Although traditionally HIIT is performed using exercise protocols with cyclical characteristics, recent studies have investigated different approaches, including HIIT using body weight (McRae et al., 2012; Evangelista et al., 2017; Machado et al., 2017; Machado et al., 2018a; Machado et al., 2018b; Rica et al., 2018; Schaun et al., 2018; Evangelista et al., 2019; Schaun et al., 2019).

Additionally, the time-efficient nature of HIIT (Gibala and McGee, 2008) and its association with bodyweight exercises (McRae et al., 2012) can facilitate the application of the exercise program at home and therefore encourage more people to perform regular physical exercise. This method is appealing, as practitioners do not need to purchase expensive equipment and the exercises can be performed anywhere and at any time. Few studies (Williams, 2008; McRae et al., 2012; Evangelista et al., 2017; Schaun and Alberton, 2020) are available in the literature that have investigated psychophysiological responses associated with HIIT using whole body exercise. In this context, Evangelista et al. (2017) shows that HIIT using whole body exercise reduced sensation of anger, depression, tension, confusion, and vigor according to the mood scale (Evangelista et al., 2017). Long term, HIIT using whole body exercise was able to increase the perception of pleasure and adherence to this exercise (McRae et al., 2012; Schaun and Alberton, 2020). However, these studies presented only one order of exercise, and the effect of exercise distribution on exercise-induced psychophysiological responses is unclear. Additionally, significant improvements have been demonstrated in cardiorespiratory fitness and neuromuscular conditioning following training programs using HIIT and body weight (Machado et al., 2018a; Schaun et al., 2019).

Interestingly, a study by Machado et al. (2018a) demonstrated that during training sessions, differences in the total number of movements and differences in heart rate were observed for exercises performed during the exercise program. The manipulation of the distribution of exercises in training sessions is not new idea, and different studies have been reported in the literature investigating different training modalities (Simão et al., 2005; Skidmore et al., 2012; Aniceto et al., 2013), however, special attention has been applied to this methodology during resistance training studies. Research has demonstrated that the number of repetitions used during exercise, and the perceived effort and energy cost of the session can be affected when the training order is changed regarding exercise distribution (Schaun and Alberton, 2020). Klika and Jordan (2013) proposed an organization of training sessions using body weight, promoting exercise using four body areas. These included total body, lower body, upper body, and core. To our knowledge, there are no studies that have investigated the influence of the distribution/order of exercise during HIIT training sessions using body weight. Therefore, the aim of the present study was to evaluate the effects of the order of exercise on training load indicators during HIIT sessions using body weight. Thus, we hypothesized that exercise distribution order will promote smaller psychophysiological responses in healthy adult men.

## 2 Material and Methods

### 2.1 Participants

Following approval from the research ethics committee of the Federal University of Espirito Santo (N°3.733.252/2019), 20 healthy cross fit practitioner men for at lest 2 year (Age: 26 ± 5 years old, body mass: 74.13 ± 12.80 kg; height: 1.71 ± 0,07 m and body mass index: 25.07 ± 2.99 kg/m^2^) voluntarily participated in the study. All procedures used followed the ethical standards of the committee on human experimentation (institutional or regional) and used guidelines outlined in the Helsinki Declaration. Signed informed consent was obtained from all participants prior to data collection. The following parameters were used as exclusion criteria: positive clinical diagnosis of diabetes mellitus, smoking, musculoskeletal complications, or cardiovascular alterations confirmed by medical evaluation and lower than 150 min of physical active per week. The Adapted International Physical Activity Questionnaire—short form (IPAQ) was used to determine the physical activity level of subjects (Matsudo et al., 2012). All participants were assigned to an exercise condition routine using a computerized random-number generator. The randomization process was completed with six subjects being assigned to different exercise blocks. Each block resulted in the allocation of two subjects to each protocol, ensuring a recruitment balance of 1:1 throughout the study. Sample size was calculated by *a priori* analysis G * Power software (v. 3.1.9.4), using a power (1—β) of 0.95, and an alpha level of 0.05.

### 2.2 Anthropometric

Height was measured using a calibrated Cardiomed (WCS model) stadiometer, with an accuracy of 115/220 cm. Measurements were performed with the cursor at an angle of 90°, with the subject maintaining a standing position with feet together in contact with the Stadiometer. Total body mass was measured by a calibrated Filizola electronic scale (Personal Line Model 150) with a 100 g scale and a maximum capacity of 150 kg. Body mass index (BMI, kg/m^2^) was calculated using the equation BMI = body mass (kg)/height^2^ (m).

### 2.3 Protocols

All participants performed three protocols each comprising of a single HIIT bodywork session, which differed regarding exercise order. The HIIT bodywork session consisted of 20 sets with 30 s of activity (TE) using “all-out” effort, followed by 30 s of passive recovery (TR). Five cycles were performed for each of the four exercises and were performed in different orders. The exercises used included jumping jack, burpee, mountain climber and squat jump.

Therefore, three designs of exercise session protocols were randomly performed according to following exercise distribution: A: jumping jack, burpee, mountain climb and squat jump); B: jumping jack, mountain climb, burpee, and squat jump) and C: burpee, squat jump, jumping jack and mountain climb.

The participants were advised not to exercise or consume any stimulants for 24 h prior to each exercise session. Each participant was instructed to consume 500 ml of water every hour in the 2 h prior to the exercise sessions. They were also advised not to consume any type of food during that period.

### 2.4 Number of Movements

The number of movements for each exercise (repetitions) performed in each set was quantified as suggested by Machado et al. (2018a).

### 2.5 Heart Rate

Heart rate (HR) was recorded continuously throughout the training session using HR monitors (Polar Electro Oy S810i, Kempele, Finland). HR data were recorded every 5 s. To reduce HR recording error during training, all subjects were asked to check their HR monitors before the session and after each set (∼ 3 and 10 min). Maximal heart rate was estimated using the Tanaka equation (Tanaka et al., 2001).

### 2.6 Blood Lactate Measurement

Capillary blood samples were taken from a sterile fingertip using a sterile lancet immediately following training sessions. The first drop of blood was discarded, and free flow blood was collected in glass capillary tubes. All blood samples used for lactate analysis were evaluated using an Accutrend® (Roche—Basel, Switzerland) as described previously (Machado et al., 2018a; Rica et al., 2018).

### 2.7 Rate of Perceived Exertion and Recovery

Subjects reported their rating of perceived exertion (RPE, scale 1–10) as described by Borg (0–10) (Foster et al., 2017), immediately at the end, and prior to each exercise set as previously outlined (Machado et al., 2018a; Machado et al., 2018b; Rica et al., 2018). Recovery was measured using a scale adapted by Laurent et al. (2011) and has been used previously in exercise studies (Machado et al., 2018a). Values on the scale ranged from 0 to 10. The closer the value 10, the greater the recovery perception of the practitioner.

### 2.8 Feeling Scale

The psychological responses to the exercise sessions were evaluated using a feeling scale instrument (Hardy and Rejeski, 1989; Frazão et al., 2016). To measure the feeling scale (FS) a 11-point bipolar scale ranging from −5 to +5 and has been used to measure affective response (pleasure/displeasure) during exercise. However, in this study the parameters were evaluated prior to and following 5 min of exercise completion. The scale range includes the following outcome measures: −5 = very bad; −3 = bad; −1 = fairly bad; 0 = neutral; +1 fairly good; +3 = good; and +5 = very good.

### 2.9 Statistical Analysis

The D’Agostino–Pearson test was applied to Gaussian distribution analysis. Two-way repeated measures ANOVA followed by Bonferroni’s post hoc test was performed considering time points and protocols as main factors to analyze responses of selected variables (heart rate, lactate, feeling scale). In addition, the comparison among exercise protocols and area under the curve was performed using a one-way repeated measures ANOVA followed by Bonferroni post hoc test for selected variables (perceived exertion, number of movements). The effect sizes (ES) based on Cohen’s d were evaluated and qualitatively interpreted using the following thresholds: < 0.2, trivial; 0.2–0.6, small; 0.6–1.2, moderate; 1.2–2.0, large; 2.0–4.0, very large and; >4.0, extremely large. An alpha of 0.05 was used to determine statistical significance. All data values were expressed as mean ± standard deviation (SD). All analyses were performed using GraphPad Prism version 6.00 for Windows (GraphPad Software, La Jolla California, United States).

## 3 Results

There were no injuries or musculoskeletal problems reported for any of the exercise sessions. All subjects completed the three training protocols. As shown in Table 1, the absolute values for heart rate and lactate concentrations significantly increased following exercise without any differences between protocols. No differences (F = 1.912, *p* = 0.1650) were found for relative heart rate between protocols (A: 93.67 ± 4.74, B: 92.70 ± 4.13, C: 94.79 ± 2.87; %).

**TABLE 1:** Absolute values of heart rate and lactate concentration according to exercise protocol.

Parameters	Before	After	95% IC of diff	ES	ANOVA		
					Effect		
					Time effect	Time*protocol	
						F	*p*
Heart rate (bpm)							
Protocol A	81.90 ± 14.13	178.05 ± 9.36*	−104.2 to −88.12	6.59	<0.0001		
Protocol B	76.95 ± 9.69	176.20 ± 8.36*	−107.3 to −91.22	7.19	<0.0001	0.67	= 0.5117
Protocol C	78.70 ± 8.71	180.20 ± 7.06*	−109.5 to −93.47	12.26	<0.0001		
Lactate (mMol.L-1)							
Protocol A	1.38 ± 0.70	13.99 ± 3.16*	−14.41 to −10.80	5.50	<0.0001		
Protocol B	1.42 ± 0.66	13.84 ± 2.99*	−14.23 to −10.62	5.74	<0.0001	0.016	= 0.9797
Protocol C	1.41 ± 0.46	13.95 ± 3.64*	−14.35 to −10.74	4.83	<0.9839		
Feeling scale							
Protocol A	4.35 ± 0.58	0.20 ± 2.07*†‡	3.11 to 5.18	2.73	<0.0001		
Protocol B	4.30 ± 0.73	−1.50 ± 2.28*‡	4.76 to 6.83	3.43	<0.0001	23.84	<0.0001
Protocol C	4.30 ± 0.80	−3.95 ± 0.88*	7.21 to 9.28	9.81	<0.0001		

Values expressed in mean ± DP, for heart rate (bpm), lactate (mMol.L-1) and feeling scale for protocol A (jumping jack, burpee, mountain climb and squat jump), protocol B (jumping jack, mountain climb, burpee, and squat jump) and protocol C (burpee, squat jump, jumping jack and mountain climb). *p < 0.0001 vs. before. ^†^ *p* < 0.0001 vs. Protocol B. ^‡^ *p* < 0.0001 vs. Protocol C.

The values for feeling scale (Table 1) revealed a significant time (F = 438.3, *p* < 0.0001) and protocol interaction (F = 28.79, *p* < 0.0001) indicating a significant reduction in this outcome measure for all protocols. However, the values observed following the sessions were significantly different between protocols. A greater reduction was observed for the B and C protocols compared to protocol A, for mean values which indicates a displeasure perception compared to protocol A.

Perceived exertion did not differ (A: 9.3 ± 0.73; B: 9.1 ± 1.21; C: 9.15 ± 1.31; F = 0.3139, *p* = 0.7062) between protocols, however, the number of movements (A: 712 ± 59, B: 524 ± 49, C: 452 ± 65; F = 107.9, *p* < 0.0001) were significantly different between all protocols, with higher values for protocol A, followed by B and C.

**FIGURE 2:**
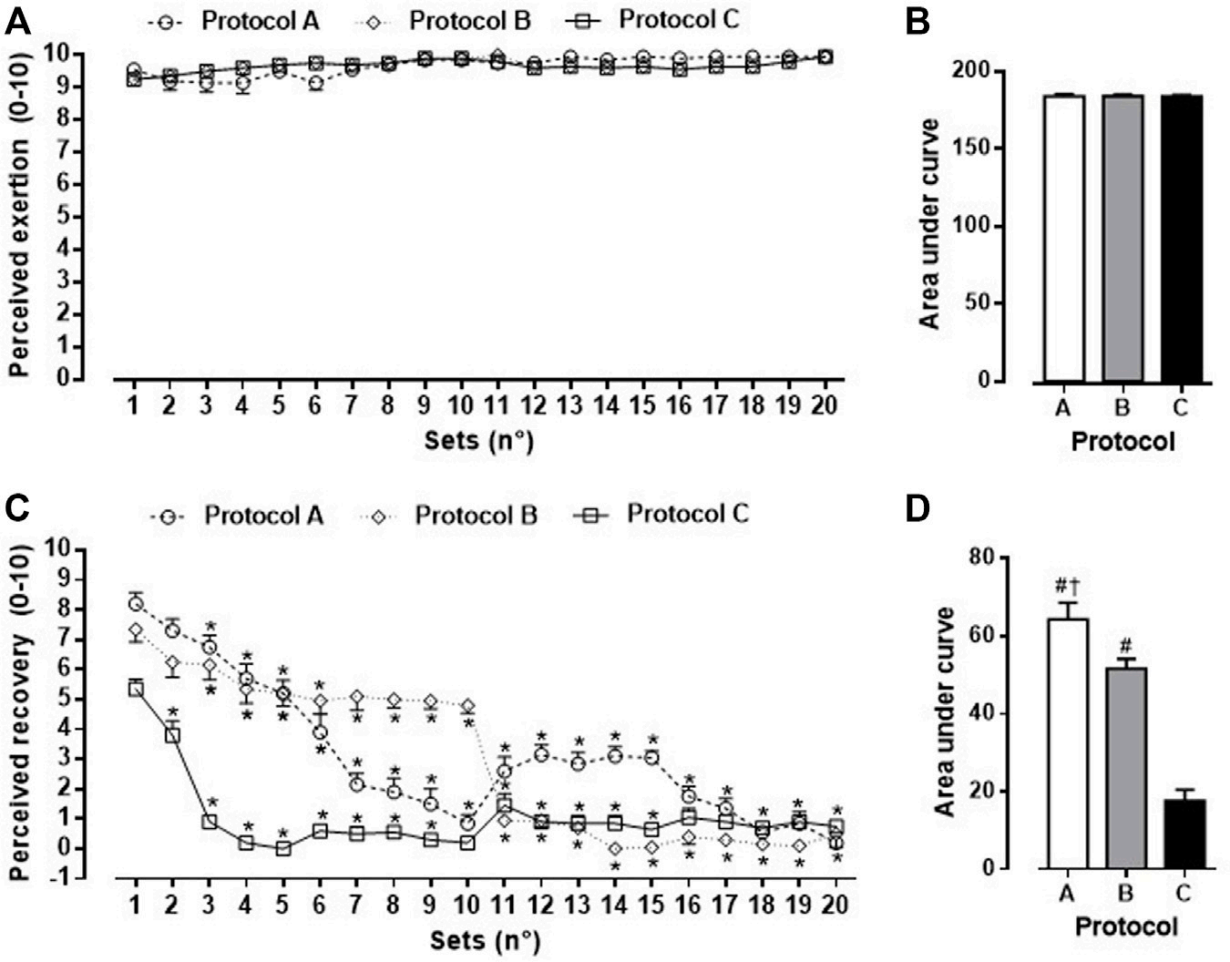
Values expressed as mean ± DP for perceived exertion around set [Panel **(A)**], area under curve for perceived exertion [Panel **(B)**], perceived recovery around set [Panel **(C)**] and area under curve for perceived exertion [Panel **(D)**] for protocol A (jumping jack, burpee, mountain climb and squat jump), protocol B (jumping jack, mountain climb, burpee, and squat jump) and protocol C (burpee, squat jump, jumping jack and mountain climb). *p < 0.001 vs. First set. ^#^ *p* < 0.001 vs. protocol C ^†^ *p* < 0.001 vs. protocol B.

Perceived exertion and recovery values are presented in Figure 2. As shown in Panel B no differences (F = 0.0074; *p* = 0.9926) were found for the area under curve for perceived exertion between protocols (A: 184 ± 6, B: 184 ± 5, C: 184 ± 5). The values for perceived recovery (Panel C) significantly differed (*p* < 0.001) from the third set compared to protocol A and B. Protocol C differed from the second set compared to first set (*p* < 0.001). Statistical differences (F = 49.42; *p* < 0.00010) were found for the area under curve (A: 64 ± 19 > B: 52 ± 11 > C: 17 ± 13) for perceived recovery (Panel D) between protocols.

## 4 Discussion

The main findings from this study demonstrated that, although there were no differences in HR, Lactate and RPE, the number of movements, the perception of recovery and pleasure were modified between protocols. To our knowledge, studies investigating the influence of exercise distribution using HIIT sessions in conjunction with body weight are few in the scientific literature. The results presented here are original and innovative and demonstrate physiological differences using variations in exercise order.

In the present study, HR did not differ between protocols. This suggests that the use of different exercise orders induced similar physiological stress and could be considered as a HIIT session. This is particularly true when values recorded during exercise were above 85% of the HR as recommended by Karvonen and Vuorimaa (1988). This has also been observed in other HIIT sessions (Maclnnis and Gibala, 2017) when subjects exercised with or without equipment (McRae et al., 2012; Gist et al., 2015). Considering HR, Machado et al. (2018a) demonstrated that exercise type induced modifications in HR for the same session. It is worth noting here, that in the present study, the same exercises were used for all sessions, varying only in temporal order. Additionally, even if performed using different exercise distributions (protocols A, B, and C) the HR values remained similar regardless of the protocol used.

**FIGURE 1:**
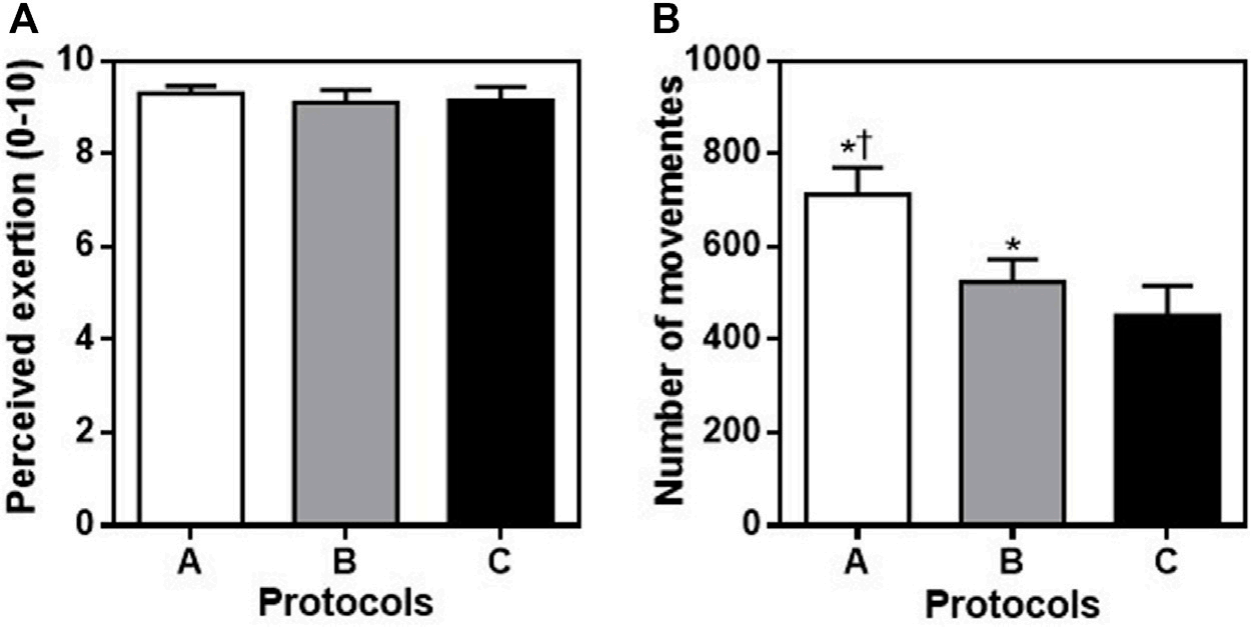
Values expressed at mean ± DP for perceived exertion [Panel **(A)**] and number of movements [Panel **(B)**] for protocol A (jumping jack, burpee, mountain climb and squat jump), protocol B (jumping jack, mountain climb, burpee, and squat jump) and protocol C (burpee, squat jump, jumping jack and mountain climb). *p < 0.001 vs. protocol C ^†^ *p* < 0.001 vs. protocol B.

In relation to blood lactate concentrations and perceived exertion, our findings agree with previous studies (Machado et al., 2018a; Machado et al., 2018b). As outlined in Table 1 (lactate) and shown in Figure 1A (perceived exertion) all protocols induced increases in these parameters following HIIT using whole body exercise. In conjunction with the HR data, it is possible to consider that the physiological stress was similar between protocols performed in this study. However, it is worth mentioning that studies using HIIT and bodyweight use maximal intensities of exercise as protocol standards (Machado et al., 2018a). As a result, it is possible that the perceived effort of programs using these characteristics are considered as severe exercise intensities.

Although there are no studies dedicated to investigating different effects of exercise distribution on programs using HIIT using bodyweight, some studies (Skidmore et al., 2012; Aniceto et al., 2013) have investigated the effects of different exercise session formats. Skidmore et al. (2012) evaluated the responses of HR and lactate concentrations to three exercise programs using different orders in the distribution of exercises. The research group varied circuit training with collective gymnastics, cycle ergometer and sprints using maximal intensities. The findings of the study demonstrated that HR, lactate concentrations and perceived exertion were higher during circuit training sessions followed by sprints. These findings agree with Aniceto et al. (2013) that training session organization can promote different physiological responses, and that the exercises have different physiological characteristics, including motor and/or metabolic differences.

One of the parameters that can influence physiological responses to high-intensity exercise sessions is the interval between series (Buchheit et al., 2009; Driller et al., 2009; Germano et al., 2022). In our study, we did not evaluate the influence of the intervals, but we did investigate the impact that the different exercise protocols had on the perception of recovery. This parameter can be interesting, especially when the purpose is to evaluate the repercussions of both the training session and the series regardless of the type/modality of HIIT. The findings of the present study were different from the findings of Machado et al. (2018a), who demonstrated a continuous reduction in the perception of recovery.

In this study, the distribution of the exercises influenced the perception of recovery, with alternating protocols inducing a greater perception of recovery throughout the training sessions. This may be explained by the greater fatiguing effect that the burpee and squat jump exercises induce regardless of when they are performed.

The exercise order may explain the differences in the total number of movements observed between protocols, especially the influence of exercises on the perception of recovery. The evaluation of different training program strategies and external load parameters is not new (Sforzo and Touey, 1996; Simão et al., 2005; Schaun et al., 2019). Our results, using body weight exercise, present similar results compared to traditional strength training, indicating that the exercise sequence or training modalities can promote alterations in exercise performance in one session. This effect of exercise order during training sessions needs consideration and is an important factor when designing programs. Training session design needs to consider if the order of the exercises used are specific to meet the training goals of a program.

Considering the perception of pleasure, studies have shown favorable (Oliveira et al., 2018; Olney et al., 2018) or unfavorable (Frazão et al., 2016; Evangelista et al., 2017; Follador et al., 2018) outcomes when using high intensity exercise. It has been proposed that the intermittent nature of interval training induces a “rebound effect” that generates a better feeling of pleasure (Jung et al., 2014). Frazão et al. (2016) demonstrated a reduction in the perception of pleasure throughout a training series in active individuals and displeasure in insufficiently active individuals performing 10 sets of exercise from 60 s to 90% of vVO2max with intervals of 60 s to 30% vVO2max. Using body weight, Evangelista et al. (2017) studied twenty-six healthy recreationally active adult men performing 8 sets of 20 s of maximal intensity exercise with 10 s of passive recovery using HIIT whole body weight. The results of the study found a reduction in the perception of pleasure until the sixth series and displeasure in the seventh and eighth grades. To our knowledge, the information on the perception of pleasure in HIIT exercise programs using body weight is still inconclusive. The findings of the present study agree with the findings of Evangelista et al. (2017) and show a reduction in the sensation of pleasure following completion of the protocol. Additionally, Schaun and Alberton (2020) suggest that whole-body interval training can be used as an enjoyable low-cost alternative to traditional treadmill-based sprint interval training (SIT) and moderate-intensity continuous training (MICT).

It is worth mentioning that the parameters related to protocols B and C exhibited displeasure after the exercise sessions. An explanation for this finding may be related to the possibility of reduced or insufficient recovery time for protocols B and C induced by the exercise distribution order. In support of this suggestion, studies have (Nogueira et al., 2012; Dalamitros et al., 2016) observed that short periods of recovery during high intensity exercise, generate residual fatigue, which contributes to the decrease in the sensation of pleasure. The perception of pleasure is crucial in adhering to physical activity programs, since the feelings experienced during exercise are reliable predictors for future participation in structured exercise. Our findings indicate that the selection of exercises during HIIT sessions using body weight can affect the perception of pleasure and therefore the possibility of adhering to the practice of HIIT.

There are some important limitations in this study. The sample size was limited to healthy and cross fit practitioner men who had experience using whole body in exercise sessions. As a result, our findings cannot be applied to overweight/obese or untrained individuals. A maximal test may also be needed to confirm the % HR kinetics during the exercise sessions. In general, HR is presented as HRminimum, HRmaximal, HRmean %HRpeak during the session. This method of presentation makes it difficult to quantify the training load by HR. Additionally, there is a large variety of HIIT applications and exercise regimes, and the results from this study cannot be applied to other forms of exercise designs and taken together, these observations limit the generalization of the results.

Although some limitations are present in this study, some positive practical applications can be observed. The organization of exercises alternately promoted improvements in the perception of recovery and pleasure in relation to other models of exercise selection. This can contribute to the development of different training strategies. The results of the present study demonstrate that protocol A produced a greater number of movements and better perception of recovery compared to protocols B and C. Additionally, protocol A induced just reduction of feeling of pleasure, differently of B and C protocol that induced displeasure. This is concerning given that these responses may promote negative exercise experiences, which may impact on exercise adherence (Williams, 2008). In this context, the use of exercise distributed alternately during HIIT-body work with “all out” efforts might favor a better performance in addition to more positive affective responses. This suggestion seems to promote a more favorable exercise experience. However, the different exercise orders used during the HIIT sessions using body weight did not alter the classical parameters of physiological responses, and the corresponding measures of HR, lactate concentrations and perceived exertion.

## Data Availability

The original contributions presented in the study are included in the article/supplementary material, further inquiries can be directed to the corresponding author.
